# Effects of *Lactobacillus paracasei* LK01 on production performance, egg quality, antioxidant capacity, immunity and intestinal health of laying hens

**DOI:** 10.3389/fvets.2025.1713771

**Published:** 2026-01-07

**Authors:** Tianjun Wu, Weixin Liu, Xinyu Yin, Linkun Zhang, Hong Chen, Ye Zhang, Cheng Zhang, Runsheng Jiang, Xiaoling Ding

**Affiliations:** College of Animal Science and Technology, Anhui Agricultural University, Hefei, China

**Keywords:** egg quality, intestinal microbiota, *Lactobacillus paracasei*, laying hens, production performance

## Abstract

This study aimed to evaluate the effects of dietary supplementation with *Lactobacillus paracasei* LK01 on production performance, egg quality, serum biochemical parameters, antioxidant capacity, inflammatory factors, intestinal digestive enzyme activities, and cecal microbiota in laying hens, while determining the optimal supplementation level. A total of 288 healthy 20-week-old Jinghong No. 1 laying hens, with uniform body weight and similar egg production rates, were stratified-randomly assigned to four groups (4 replicates/group, 18 hens/replicate): the control group (CON; basal diet) and the low-level (LLP), medium-level (MLP), and high-level (HLP) groups, which received the basal diet supplemented with 10⁶, 10^7^, and 10^8^ CFU/kg of *L. paracasei* LK01, respectively. The trial included a 1-week adaptation period followed by an 8-week experimental period. Results indicated: (1) *L. paracasei* LK01 supplementation increased average egg weight and significantly enhanced egg production rates in the MLP and HLP groups compared to the CON group (*p* < 0.05); (2) The HLP group exhibited increased albumen height and Haugh units, while the LLP group showed improved eggshell strength (*p* < 0.05); (3) Total protein (TP) levels were elevated in all supplemented groups (*p* < 0.05), with albumin (ALB) levels increased in the LLP and MLP groups, and total cholesterol (TC) and triglyceride (TG) levels decreased (*p* < 0.05); (4) Total antioxidant capacity (T-AOC) was enhanced in all supplemented groups, and serum IL-6 and TNF-*α* levels were reduced in the HLP group (*p* < 0.05); (5) No significant differences were observed in intestinal digestive enzyme activities (duodenum, jejunum, ileum) between groups (*p* > 0.05); At the phylum level, the abundance of *Spirochaetota* was decreased in all supplemented groups (*p* < 0.05), with *Bacteroidetes* and *Firmicutes* remaining the dominant phyla. At the genus level, the abundances of *Fusobacterium* and *Treponema* were reduced in all supplemented groups (*p* < 0.05). In conclusion, dietary *L. paracasei* LK01 improved laying hen performance, egg quality, serum biochemical parameters, antioxidant capacity, and reduced pro-inflammatory factors while suppressing harmful gut microbiota. The 10^8^ CFU/kg dose appeared optimal based on results; However, as this was the highest concentration tested, further research is needed to confirm the precise optimal level.

## 1 Introduction

Laying hen farming serves as a vital component of the global livestock industry, providing a high-quality protein source for humans. With growing consumer concerns regarding food safety, animal welfare, and environmental sustainability, laying hen production faces multiple challenges. These include enhancing performance, optimizing egg quality, and minimizing environmental pollution (1). Probiotics, defined as live microorganisms that confer health benefits to the host when administered in adequate amounts (2), have been shown to significantly improve poultry performance and health by modulating intestinal microbiota, enhancing immune function, and improving nutrient absorption (3–6). In laying hens, probiotic supplementation has been demonstrated to increase egg-laying rates, improve eggshell quality, reduce yolk cholesterol content, and enhance antioxidant capacity (7–9).

*Lactobacilli* represent a key category of probiotic bacteria, noted for their acid-producing fermentation capabilities and intestinal health-promoting effects (10, 11). *Lactobacillus paracasei*, a common lactic acid bacterium prevalent in fermented foods and animal intestines, exhibits favorable probiotic properties (12, 13). Research indicates that *L. paracasei* demonstrates strong efficacy in antibacterial (14, 15), antioxidant (16, 17), as well as in preserving gut microbiota equilibrium (18, 19) and modulating immune responses (20, 21). Liu et al. (22) reported that supplementing the basal diet with *L. paracasei* LK01 enhanced growth performance and digestive enzyme activity in broiler chickens, while also improving serum antioxidant capacity, immune function, intestinal health, and digestive enzyme activity. In laying hens, Joo et al. (23) demonstrated that supplementation with *L. paracasei* NSMJ56 increased egg weight and Haugh units, and augmented the antioxidant capacity of the intestinal mucosa. Although existing studies support the benefits of *L. paracasei* in improving animal production performance and health, its application in livestock and poultry production remains limited. The efficacy and applicability of the specific strain *L. paracasei* LK01 under diverse rearing conditions require further systematic validation. Therefore, this study aimed to investigate the impacts of *L. paracasei* LK01 on production performance, egg quality, serum antioxidant capacity, immunity, intestinal digestive enzyme activities, and cecal microbiota in laying hens. The goal was to provide a scientific foundation for precision farming and probiotic application in poultry production.

## 2 Materials and methods

### 2.1 Institutional review board statement

This experiment was approved by the Experimental Animal Welfare and Ethics Committee of the College of Animal Science and Technology, Anhui Agricultural University (Approval No. SYDW-P20210900601).

### 2.2 Bacterial strains

The bacterial strain used was *L. paracasei* LK01, which has been deposited in the China General Microbiological Culture Collection Center (CGMCC, Accession No. 33033).

### 2.3 Birds, experimental design and diets

A total of 288 Jinghong No. 1 laying hens, aged 20 weeks with an egg-laying rate of 83 ± 2% and body weight of 1.65 ± 0.10 kg, were allocated to four groups using a stratified randomization method, with four replicates per group and 18 hens per replicate: the control group (CON; basal diet) and the low-level (LLP), medium-level (MLP), and high-level (HLP) groups, supplemented with 10⁶, 10^7^, and 10^8^ CFU/kg of *L. paracasei* LK01 in the basal diet, respectively. According to the method of Liu (22), lyophilized *L. paracasei* LK01 powder (viable bacterial count ≥2.0 × 10^9^ CFU/g) was prepared and incorporated into the basal diet at 1 g/kg to formulate experimental diets with varying bacterial concentrations. During the trial, all hens were subjected to restricted feeding. Following a 7-day adaptation period, daily feed intake was established at 123 g per hen. The experimental period spanned 8 weeks.

The experiment was carried out at Dongshan Breeding Base of Anhui Academy of Agricultural Sciences, using three-layer three-dimensional cage culture, the experimental season was winter, the temperature of the chicken house was maintained at 15 ± 1 °C, the relative humidity was 55–65%, and the natural ventilation was combined with the mechanical ventilation system; The light management was carried out under the system of 16 L:8D (05:00-21:00 for light), and the intensity of the artificial supplemental light was 15–20 lux. The ration feeding (123 g/each/day) was performed at 08:00 and 15:00 every day, residual feed was weighed weekly and corrected for spillage to calculate actual intake. Free drinking clean water provided by nipple type water supply system. The birds were routinely disinfected weekly and immunized against Newcastle Disease and Avian Influenza according to standard procedures for laying hens. The number of eggs laid and egg weight were recorded daily to calculate the egg production rate, average egg weight and feed-to-egg ratio. The basal ration was formulated with reference to the agricultural industry standard of the People’s Republic of China – Chicken Feeding Standard (NY/T 33-2004) and modified according to the production practice, and its nutrient composition is shown in Table 1.

**Table 1:** Composition and nutrient concentrations of basic diets (air dried basis).

Ingredients	Proportions(%)	Nutrient Index	Content Value
Corn	62.00	ME, MJ/kg^2^	11.50
Soybean meal	22.00	CP, %	16.50
Wheat bran	5.00	EE, %	2.80
Limestone	6.00	CF, %	3.50
Dicalcium phosphate	1.50	Ash, %	4.00
NaCl	0.30	Methionine, %	0.35
Vegetable oil	1.00	Lysine, %	0.75
Premix^1^	1.20	Ca, %	3.75
DL-methionine	0.15	TP, %	0.38
L-lysine HCl	0.10		
Total	100.0		

^1^The premix provided the following for per kg of basal diet: VA 11.86 KIU, VD3 5.85 KIU, VE 98.04 mg, VK3 7.38 mg, VB1 6.31 mg, VB2 12.32 mg, VB6 8.71 mg, VB12 0.05 mg, Niacin 94.90 mg, Calcium pantothenate 16.30 mg, Folic acid 3.075 mg, Biotin 0.24 mg, Antioxidant 10.99 mg, Cu 3.29 g, Fe 21.61 mg, Zn 29.51 mg, Mn 39.92 mg, Ca 0.46 mg, Na 0.12 mg, Co 0.009 mg. ^2^ME was a calculated value, while the others were measured values. Nutrient contents were analyzed on an air-dried basis (moisture content: 10.5%) using AOAC International methods.

### 2.4 Sample collection

All sample collection was completed within 24 h prior to the experiment’s conclusion. Two fresh eggs (laid within 24 h, free of cracks or deformities) were randomly selected from each replicate, yielding a total of 32 eggs for egg quality assessment. Blood samples were collected after a 12-h fasting period; eight laying hens per group were randomly selected, and 8 mL of whole blood was aseptically drawn from the wing vein into non-anticoagulated vacuum blood collection tubes. These tubes were tilted and incubated at room temperature for 30 min, then centrifuged at 3,500 rpm for 10 min to separate serum, which was stored at −20 °C. Blood samples were simultaneously collected from four hens per replicate. Concurrently, intestinal samples were obtained from eight randomly selected hens per group (two per replicate) to ensure representation across replicates: 4 cm segments of the duodenum, jejunum, and ileum were aseptically excised, rinsed with PBS buffer, and mucosal tissues were scraped. Approximately 0.5 g of cecal contents was collected from the distal cecum (2 cm from the end). All intestinal samples were placed in cryopreservation tubes, immediately snap-frozen in liquid nitrogen, and stored at −80 °C in an ultra-low-temperature freezer.

### 2.5 Measurement of production performance

During the experimental period, the number of eggs laid and their weights were recorded daily for each replicate to calculate the average egg weight (AEW) as total egg weight divided by the number of eggs laid, and the average daily laying rate (ADLR) as (total number of eggs laid during the statistical period/(number of hens housed × number of days)) × 100%. Residual feed was weighed daily to determine the average daily feed intake (ADFI) as total feed consumption divided by (number of days × number of hens), and the feed-to-egg ratio (F/E) as feed consumption divided by total egg weight. Egg weight, albumen height, and Haugh units were measured using a multifunctional egg quality analyzer (EMT-5200, Robotmation Co., Ltd., Japan). Eggshell strength was evaluated with an eggshell strength gage (EGG-0503, Robotmation Co., Ltd., Japan). Eggshell thickness was determined using an eggshell thickness meter (TI-PVX, ORKA Food Technology Ltd., Israel), with measurements taken at the blunt end, pointed end, and middle of each egg using an electronic vernier caliper to calculate the egg shape index.

### 2.6 Measurement of serum biochemical indices

Serum indexes were measured using a Myeri BS-380 automatic biochemical analyzer (Shenzhen Myeri Biomedical Electronics Co., Ltd., Shenzhen, China) to determine the levels of alanine aminotransferase (ALT), aspartate aminotransferase (AST), total protein (TP), albumin (ALB), urea nitrogen (BUN), total cholesterol (TC), triglycerides (TG), calcium (Ca), and phosphorus (P).

### 2.7 Measurement of serum immunity and antioxidant indices

Serum immunoglobulin (IgA, IgG, IgM), antioxidant indices total antioxidant capacity (T-AOC), malondialdehyde (MDA), total superoxide dismutase (T-SOD), and glutathione peroxidase (GSH-Px), and inflammatory cytokines (IL-1β, IL-2, IL-6, and TNF-*α*) were quantified using commercial enzyme-linked immunosorbent assay (ELISA) kits from Nanjing Jiancheng Bioengineering Institute (Nanjing, China; Cat. Nos. H108-1-2, H106-1-1, H109-1-1, A015-2, A003-1, A001-1, A005-1, H002-1, H003-1, H007-1, and H009-1, respectively). All procedures were performed strictly according to the manufacturer’s instructions.

### 2.8 High-throughput sequencing of intestinal flora

A total of 16 cecal content samples (4 samples per group, with each sample collected from a different replicate) were immediately snap-frozen in liquid nitrogen after collection and shipped to Shanghai Lingen Biotechnology Co., Ltd. for 16S rRNA gene amplicon sequencing. Microbial genomic DNA was extracted using the E. Z. N. A.® Soil DNA Kit(catalog number: D5625-01, Omega Bio-tek, Norcross, GA, USA). Negative controls (no-template controls) were included during DNA extraction and PCR to monitor contamination. Post-extraction, DNA quality was assessed using NanoDrop spectrophotometry (A260/280 > 1.8; A260/230 > 2.0; model: NanoDrop 2000, Thermo Fisher Scientific, Waltham, MA, USA) and Qubit 4.0 fluorometry (Thermo Fisher Scientific, Waltham, MA, USA), with integrity confirmed by 1% agarose gel electrophoresis. DNA concentrations were normalized to 5–10 ng/μL using TE buffer. The V3-V4 hypervariable region was amplified by PCR with the 338F/806R primer pair using the TransStart FastPfu DNA Polymerase kit (catalog number: AP221-02, TransGen Biotech, Beijing, China). PCR conditions were: initial denaturation at 95 °C for 3 min; 27 cycles of 95 °C for 30 s, 55 °C for 30 s, 72 °C for 45 s; final extension at 72 °C for 10 min. Sequencing libraries were constructed using the Nextera XT DNA Library Preparation Kit (catalog number: FC-131-1096, Illumina, San Diego, CA, USA) and subjected to paired-end sequencing (2 × 250 bp) on the Illumina MiSeq platform. Raw reads were quality-filtered using fastp software (version 0.20.0) (24), assembled with assembled with FLASH (version 1.2.7; minimum overlap 10 bp, maximum mismatch rate 0.2) (25), and clustered into operational taxonomic units (OTUs) at 97% sequence similarity using UPARSE (version 7.1) (26, 27). Taxonomic classification was performed for each sequence using the RDP Classifier (version 2.2) (28) against the Silva 16S rRNA database (v138), with a confidence threshold of 70%. Alpha diversity indices (ACE, Chao1, Shannon, and Simpson) were computed using QIIME2 (v2023.9) based on rarefied OTU tables (depth: 10,000 reads/sample) to account for sequencing depth variations, and inter-sample differences in species composition were analyzed at various taxonomic levels. The 16S rRNA sequencing data for all the samples were deposited into the NCBI Sequence Read Archive (SRA) under accession number PRJNA1313457 (http://www.ncbi.nlm.nih.gov/bioproject/1313457, accessed on 2 September 2025).

### 2.9 Measurement of intestinal digestive enzyme activity

Commercial enzyme-linked immunosorbent assay (ELISA) kits (Nanjing Jiancheng, Institute of Bioengineering, Nanjing, China; Cat. Nos. A080-2 for protease, A054-2 for amylase, and A054-1 for lipase) were used to determine the activities of protease, amylase, and lipase in the duodenal, jejunal, and ileal mucosa. All assays were performed strictly according to the manufacturer’s instructions.

### 2.10 Statistics and analysis of data

Data were expressed as mean ± standard error (SEM). Each replicate cage (18 hens) served as the experimental unit (*n* = 4 replicates per treatment). Data normality and homogeneity of variance were checked using the Shapiro–Wilk test and Levene’s test, respectively. All data satisfied parametric assumptions. The test results were statistically analyzed by one-way analysis of variance (ANOVA) using SPSS v26.0 software, and Tukey’s test was used to compare the significance of multiple data, with *p* < 0.05 was considered significant. Graphs were generated using GraphPad Prism version 8.0.

## 3 Results

### 3.1 Production performance

As shown in Table 2, the average daily laying rate (ADLR) was significantly higher in the MLP and HLP groups compared with the CON group (*p* < 0.05). Average egg weight was significantly increased across all experimental groups (*p* < 0.05), with the HLP group exhibiting a significantly greater increase than the LLP and MLP groups (*p* < 0.05). Additionally, the feed-to-egg ratio was reduced in all experimental groups, with a significant reduction observed in the HLP group (*p* < 0.05).

**Table 2:** Effects of *L. paracasei* LK01 on performance of production in laying hens.

Items	CON	LLP	MLP	HLP	*p*-value
ADFI,g/d	122.25 ± 1.36	123.01 ± 2.01	122.78 ± 1.89	123.78 ± 3.11	0.851
ADLR/%	84.11 ± 1.05^c^	85.89 ± 0.49^bc^	86.73 ± 0.72^b^	90.56 ± 0.58^a^	<0.001
AEW/g	51.31 ± 0.75^c^	53.64 ± 0.52^b^	53.92 ± 0.41^b^	56.97 ± 0.45^a^	<0.001
F/E	2.38 ± 0.04^a^	2.29 ± 0.02^ab^	2.28 ± 0.03^ab^	2.17 ± 0.02^b^	0.004

CON, control, basal diet; LLP, basal diet + 10^6^ CFU/kg *L. paracasei* LK01; MLP, basal diet + 10^7^ CFU/kg *L. paracasei* LK01; HLP, basal diet + 10^8^ CFU/kg *L. paracasei* LK01. Different letters in the same row indicate significant differences (*p* < 0.05). Results are expressed as mean ± standard error of the mean (SEM). *n* = 8.

### 3.2 Egg quality

As shown in Table 3, dietary supplementation with *L. paracasei* LK01 had no significant effect on egg shape index or eggshell thickness compared with the CON group (*p* > 0.05). Albumen height and Haugh units were significantly increased in the HLP group (*p* < 0.05), with no significant changes observed in the LLP and MLP groups. Egg weight was significantly higher in the HLP group (*p* < 0.05), with an increasing trend noted in the LLP and MLP groups. Additionally, eggshell strength was significantly greater in the LLP group compared with the CON group (*p* < 0.05).

**Table 3:** Effects of *L. paracasei* LK01 on egg quality in laying hens.

Items	CON	LLP	MLP	HLP	*p*-value
Albumen height/mm	7.06 ± 0.31^b^	7.14 ± 0.27^ab^	7.36 ± 0.38^ab^	8 ± 0.30^a^	<0.001
Haugh unit	83.98 ± 1.95^b^	84.02 ± 1.90^ab^	84.88 ± 1.72^ab^	87.92 ± 1.60^a^	<0.001
Egg weight/g	59.20 ± 1.66^b^	60.34 ± 0.68^ab^	62.98 ± 4.96^ab^	65.80 ± 1.38^a^	<0.001
Eggshell Strength/N	4.28 ± 0.78^b^	4.77 ± 0.12^a^	4.66 ± 0.45^ab^	4.49 ± 0.38^ab^	0.011
Egg Shape Index	1.27 ± 0.01	1.30 ± 0.11	1.31 ± 0.20	1.31 ± 0.04	0.607
Eggshell thickness/mm	0.336 ± 0.005	0.368 ± 0.010	0.369 ± 0.006	0.357 ± 0.004	0.134

CON, control, basal diet; LLP, basal diet + 10^6^ CFU/kg *L. paracasei* LK01; MLP, basal diet + 10^7^ CFU/kg *L. paracasei* LK01; HLP, basal diet + 10^8^ CFU/kg *L. paracasei* LK01. Different letters in the same row indicate significant differences (*p* < 0.05). Results are expressed as mean ± standard error of the mean (SEM). *n* = 8.

### 3.3 Serum biochemical indicators

As shown in Table 4, no significant differences in serum AST, BUN, or Ca levels between the experimental groups (LLP, MLP, HLP) and the CON group (*p* > 0.05). ALT levels were significantly elevated in the HLP group (*p* < 0.05), and TP levels were significantly increased across all experimental groups (*p* < 0.05). Notably, ALB levels were significantly higher in the MLP and HLP groups compared with the CON and LLP groups (*p* < 0.05), while TC levels were significantly lower in these groups (*p* < 0.05). TG levels were significantly reduced in all experimental groups relative to the CON group (*p* < 0.05), and *p* levels were significantly elevated in the MLP group compared with the CONgroup (*p* < 0.05).

**Table 4:** Effects of *L. paracasei* LK01 on serum biochemical indices in laying hens.

Items	CON	LLP	MLP	HLP	*p*-value
AST(U/L)	23.16 ± 0.64	25.92 ± 2.69	30.59 ± 2.94	27.42 ± 3.62	0.175
ALT(U/L)	20.00 ± 2.00^b^	24.50 ± 1.89^ab^	25.16 ± 2.51^ab^	28.65 ± 1.11^a^	0.016
TP(g/L)	83.58 ± 1.34^c^	105.37 ± 2.23^a^	92.16 ± 3.75^b^	90.44 ± 3.10^b^	<0.001
ALB(g/L)	41.36 ± 0.62^a^	43.54 ± 1.22^a^	45.48 ± 1.14^b^	48.08 ± 0.62^b^	0.001
BUN(mmol/L)	4.98 ± 0.53	4.94 ± 0.18	5.12 ± 0.48	5.19 ± 0.53	0.879
TC (mmol/L)	4.05 ± 0.25^a^	3.29 ± 0.88^ab^	2.52 ± 0.21^b^	2.32 ± 0.37^b^	0.005
TG (mmol/L)	1.57 ± 0.03^a^	1.19 ± 0.12^b^	1.14 ± 0.13^b^	0.93 ± 0.09^b^	<0.001
P(mmol/L)	7.61 ± 0.47^b^	8.56 ± 0.33^ab^	8.85 ± 0.41^a^	8.39 ± 0.25^ab^	0.012
Ca(mmol/L)	3.58 ± 0.18	3.72 ± 0.39	3.57 ± 0.26	4.26 ± 0.41	0.208

CON, control, basal diet; LLP, basal diet + 10^6^ CFU/kg *L. paracasei* LK01; MLP, basal diet + 10^7^ CFU/kg *L. paracasei* LK01; HLP, basal diet + 10^8^ CFU/kg *L. paracasei* LK01. Different letters in the same row indicate significant differences (*p* < 0.05). Results are expressed as mean ± standard error of the mean (SEM). *n* = 8.

### 3.4 Serum immune and antioxidant markers

As shown in Table 5, serum T-AOC levels were significantly higher in all experimental groups (LLP, MLP, HLP) than in the CON group (*p* < 0.05), whereas MDA levels were significantly lower in the MLP and HLP groups (*p* < 0.05). Regarding inflammatory cytokines, supplementation with *L. paracasei* LK01 had no significant effect on IL-1β levels (*p* > 0.05). Notably, IL-2 levels were significantly lower in the LLP and HLP groups compared to the CON group (*p* < 0.05). Similarly, IL-6 levels were significantly reduced in the MLP and HLP groups relative to the CON group (*p* < 0.05), with the HLP group showing significantly lower IL-6 levels than the LLP group (*p* < 0.05). Additionally, TNF-*α* levels were significantly lower in the HLP group compared to both the CON and LLP groups (*p* < 0.05).

**Table 5:** Effects of *L. paracasei* LK01 on immunity and antioxidant indices in laying hens.

Items	CON	LLP	MLP	HLP	*p*-value
IgA (ng/mL)	246.73 ± 10.16	277.48 ± 11.57	271.80 ± 16.38	260.69 ± 2.23	0.225
IgG (μg/mL)	4.94 ± 0.28	5.70 ± 0.51	5.89 ± 0.32	6.09 ± 0.51	0.117
IgM (ng/mL)	309.90 ± 14.69	276.45 ± 23.70	278.42 ± 20.22	264.41 ± 20.94	0.196
T-AOC (U/L)	89.15 ± 4.27^a^	111.73 ± 2.259^b^	108.5 ± 2.48^b^	118.82 ± 2.85^b^	<0.001
GSH-Px (mIU/mL)	11.51 ± 0.83	10.62 ± 0.96	10.51 ± 0.75	12.22 ± 0.68	0.304
T-SOD (U/mL)	11.91 ± 0.86	11.62 ± 0.91	12.98 ± 1.56	11.41 ± 0.99	0.472
MDA (nmol/L)	5.33 ± 0.18^a^	5.09 ± 0.33^ab^	4.42 ± 0.16^b^	4.83 ± 0.38^b^	0.009
IL-1β (pg/mL)	178.61 ± 6.56	165.38 ± 5.13	160.64 ± 4.82	173.80 ± 7.29	0.168
IL-2 (pg/mL)	170.43 ± 7.37^a^	128.14 ± 5.14^b^	147.38 ± 8.98^ab^	141.77 ± 8.94^b^	0.003
IL-6 (pg/mL)	110.60 ± 2.75^a^	107.93 ± 4.81^ab^	97.28 ± 4.69^bc^	91.11 ± 2.85^c^	<0.001
TNF-α (pg/mL)	269.61 ± 7.64^a^	264.93 ± 10.16^a^	249.93 ± 6.52^ab^	232.55 ± 1.43^b^	<0.001

CON, control, basal diet; LLP, basal diet + 10^6^ CFU/kg *L. paracasei* LK01; MLP, basal diet + 10^7^ CFU/kg *L. paracasei* LK01; HLP, basal diet + 10^8^ CFU/kg *L. paracasei* LK01. Different letters in the same row indicate significant differences (*p* < 0.05). Results are expressed as mean ± standard error of the mean (SEM). *n* = 8.

### 3.5 Intestinal digestive enzyme activity

As shown in Table 6, the differences in the levels of duodenal, jejunal and ileal digestive enzyme activities were not significant (*p* > 0.05) in the test groups compared to the control group.

**Table 6:** Effects of *L. paracasei* LK01 on intestinal digestive enzyme activity indices in laying hens.

Items	CON	LLP	MLP	HLP	*p*-value
Duodenum					
Protease IU/mL	36.01 ± 3.76	31.26 ± 5.31	34.50 ± 1.70	33.07 ± 5.74	0.759
Amylase U/L	47.25 ± 4.28	39.88 ± 2.19	43.13 ± 1.16	36.75 ± 1.40	0.142
Lipase U/mL	27.83 ± 0.19	27.64 ± 3.25	23.79 ± 1.61	31.02 ± 2.46	0.211
Jejunum					
Protease IU/mL	30.28 ± 1.55	29.05 ± 1.82	34.52 ± 3.63	30.43 ± 2.54	0.324
Amylase U/L	42.62 ± 0.29	34.80 ± 0.50	33.64 ± 1.34	31.91 ± 2.55	0.095
Lipase U/mL	27.66 ± 2.90	34.80 ± 0.50	33.64 ± 1.34	31.91 ± 2.55	0.188
Ileum					
Protease IU/mL	30.98 ± 2.85	31.26 ± 5.31	34.50 ± 1.70	37.80 ± 1.74	0.416
Amylase U/L	43.89 ± 2.98	41.01 ± 0.99	41.45 ± 3.12	39.66 ± 1.55	0.563
Lipase U/mL	28.18 ± 0.91	27.67 ± 2.74	27.50 ± 3.52	31.91 ± 2.55	0.297

CON, control, basal diet; LLP, basal diet + 10^6^ CFU/kg *L. paracasei* LK01; MLP, basal diet + 10^7^ CFU/kg *L. paracasei* LK01; HLP, basal diet + 10^8^ CFU/kg *L. paracasei* LK01. Different letters in the same row indicate significant differences (*p* < 0.05). Results are expressed as mean ± standard error of the mean (SEM). *n* = 8.

### 3.6 Gut microbiota

**Figure 1:**
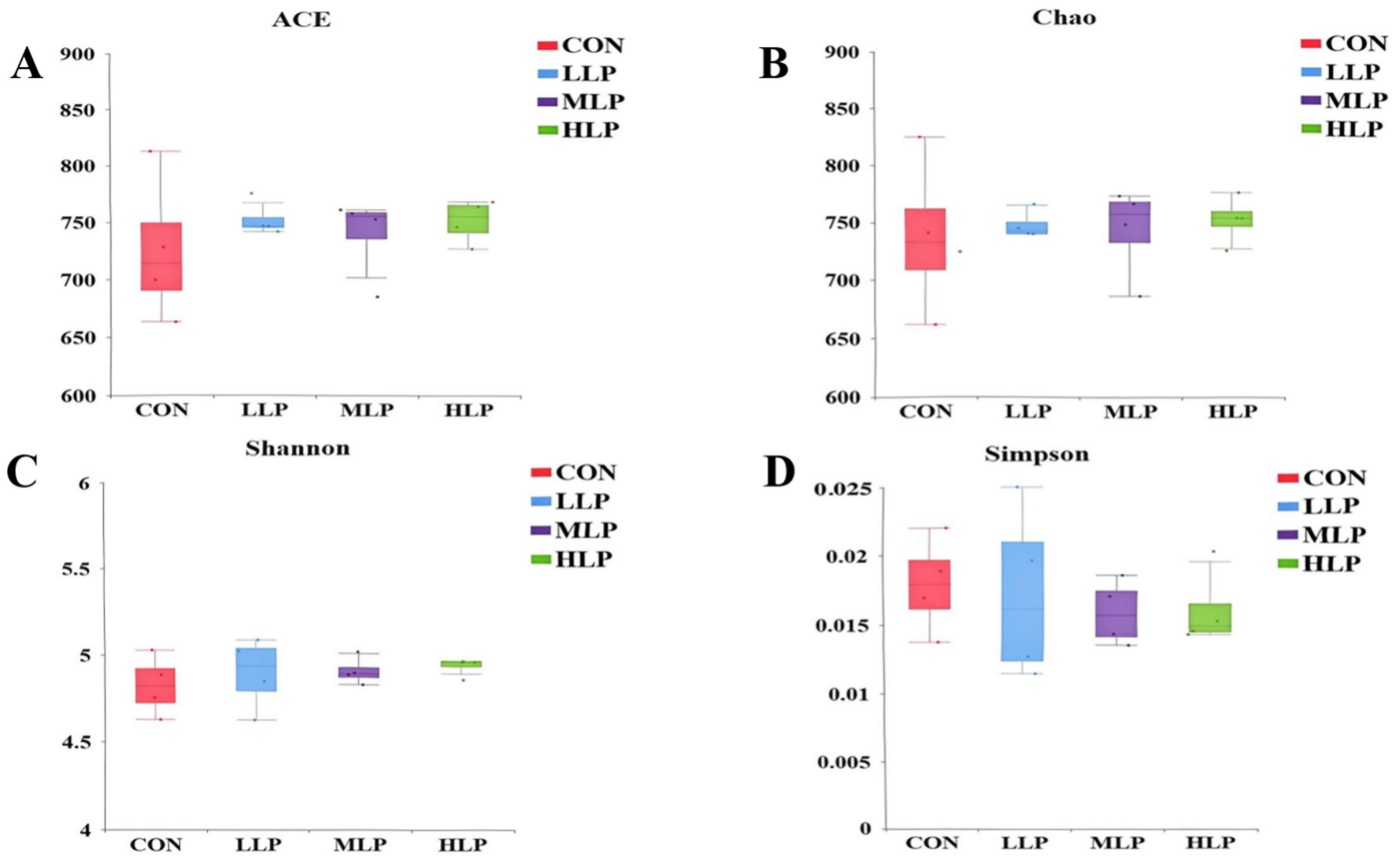
Alpha diversity analysis of the cecal microbiota. **(A)** ACE Indexes; **(B)** Chao1 Indexes; **(C)** Shannon Indexes; **(D)** Simpson Indexes.

Alpha diversity analysis of bacterial communities (Figure 1) revealed no significant differences in the Shannon, Simpson, ACE, and Chao1 indices between the experimental groups (LLP, MLP, HLP) and the CON group (*p* > 0.05).

**Figure 2:**
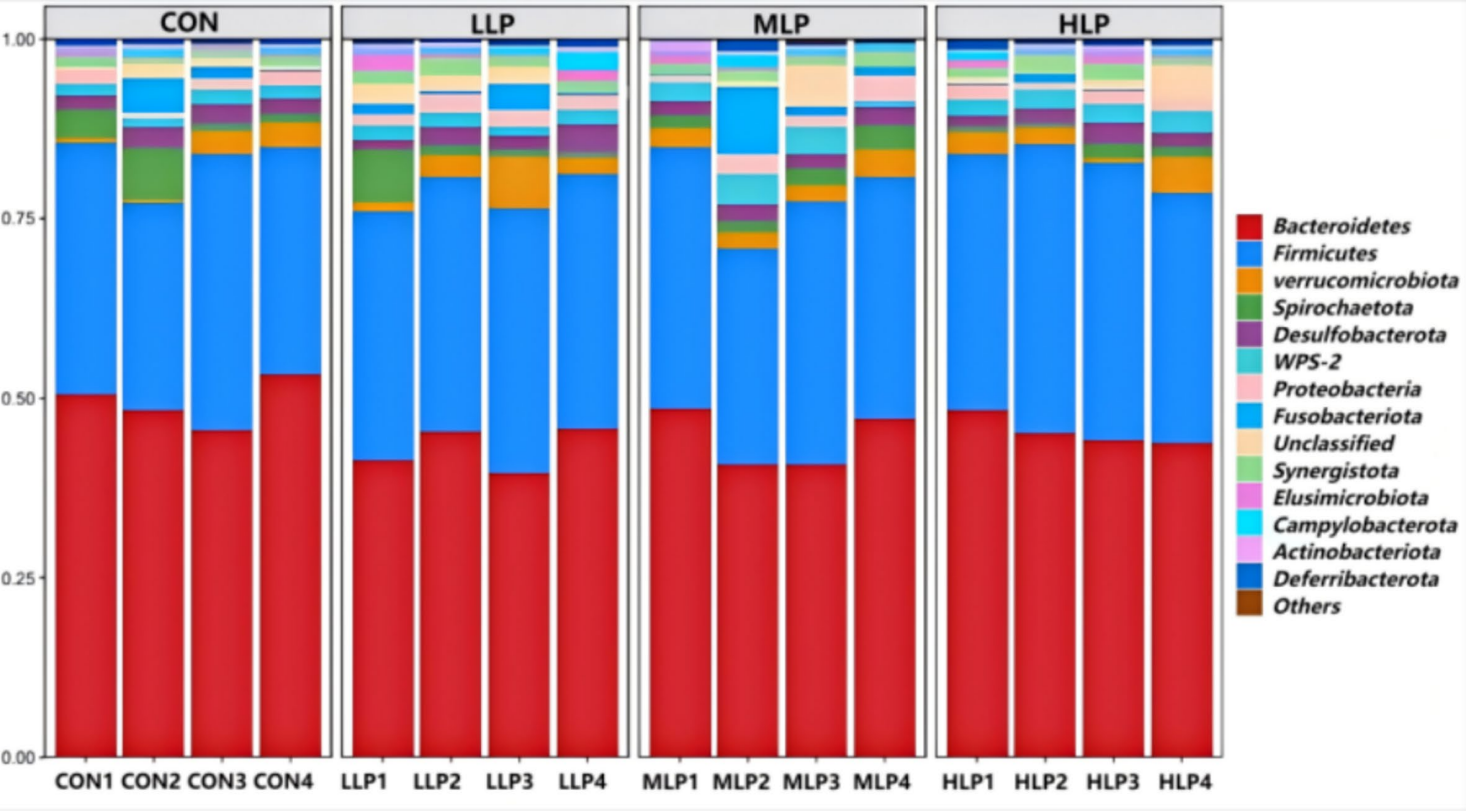
Relative abundance of phylum level in cecum microorganisms.

**Figure 3:**
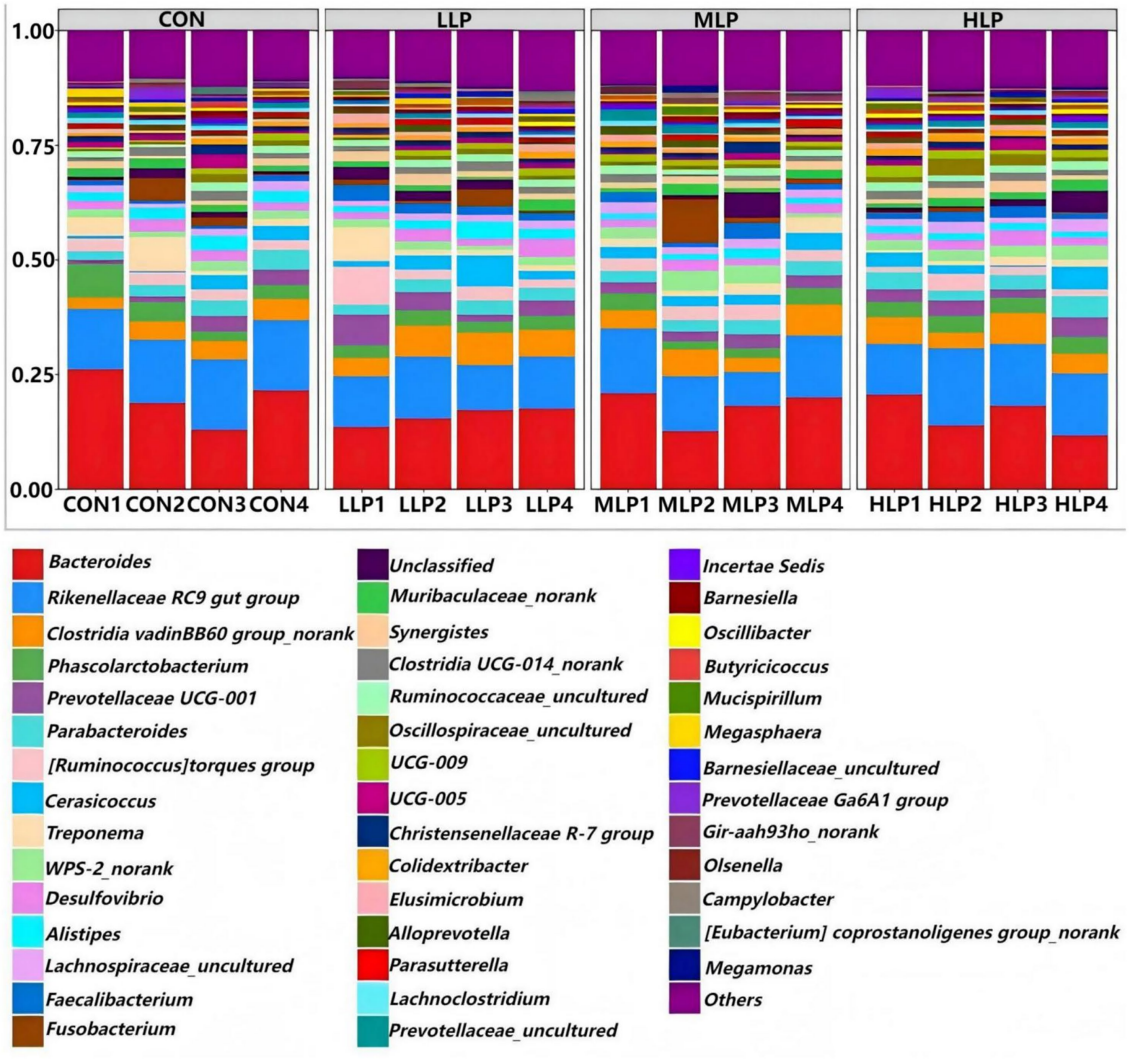
Relative abundance of genus level in cecum microorganisms.

At the phylum level (Figure 2; Table 7), the cecal microbiota of the experimental groups primarily comprised *Firmicutes*, *Bacteroidetes*, *Spirochaetota*, *Desulfobacterota*, and *Proteobacteria*, with *Firmicutes* and *Bacteroidetes* as the dominant phyla. The relative abundance of *Spirochaetota* was significantly lower in all experimental groups compared to the CON group (*p* < 0.05), with the greatest reduction observed in the HLP group. At the genus level (Figure 3; Table 8), the cecal microbiota mainly included *Bacteroides*, *Rikenellaceae RC9 gut group*, *Fusobacterium*, *Phascolarctobacterium*, *Treponema*, and *WPS-2_norank*, with *Bacteroides* and *Rikenellaceae RC9 gut group* as the dominant genera. The abundance of *Fusobacterium* was significantly reduced in the MLP and HLP groups, and that of *Treponema* was significantly reduced in all experimental groups (*p* < 0.05).

**Table 7:** Relative abundance of phylum level in cecum dominant microorganisms %.

Items	CON	LLP	MLP	HLP	*p*-value
*Bacteroidetes*	48.19 ± 1.46	44.37 ± 1.47	45.51 ± 2.33	45.91 ± 1.22	0.356
*Fimicutes*	33.51 ± 2.07	34.55 ± 0.46	34.19 ± 1.58	37.34 ± 1.58	0.289
*Spirochaetota*	4.20 ± 1.68^a^	1.15 ± 0.11^b^	1.84 ± 0.28^b^	0.98 ± 0.0.26^b^	0.004
*Desulfobacterota*	2.51 ± 0.33	2.83 ± 0.58	2.35 ± 0.21	2.36 ± 0.39	0.632
*Proteobacteria*	1.65 ± 0.13^b^	2.20 ± 0.41^ab^	2.66 ± 0.41^a^	1.74 ± 0.17^b^	0.037

CON, control, basal diet; LLP, basal diet + 10^6^ CFU/kg *L. paracasei* LK01; MLP, basal diet + 10^7^ CFU/kg *L. paracasei* LK01; HLP, basal diet + 10^8^ CFU/kg *L. paracasei* LK01. Different letters in the same row indicate significant differences (*p* < 0.05). Results are expressed as mean ± standard error of the mean (SEM). *n* = 4.

**Table 8:** Relative abundance of genus level in cecum dominant microorganisms.

Items	CON	LLP	MLP	HLP	*p*-value
*Bacteroides*	17.80 ± 2.55	16.80 ± 0.69	19.65 ± 0.79	17.70 ± 2.01	0.412
*Rikenellaceae_RC9_gut_group*	14.00 ± 0.64	11.897 ± 0.74	13.21 ± 0.68	14.62 ± 1.10	0.278
*Fusobacterium*	6.43 ± 1.20^a^	6.37 ± 0.68^a^	3.22 ± 0.85^b^	4.90 ± 0.52^b^	0.017
*Phascolarctobacterium*	3.01 ± 0.56	3.09 ± 0.73	3.23 ± 0.46	3.66 ± 0.13	0.586
*Treponema*	4.17 ± 1.68^a^	1.13 ± 0.12^b^	1.81 ± 0.30^b^	0.96 ± 0.27^b^	0.023
*WPS-2_norark*	1.69 ± 0.22	1.65 ± 0.25	2.96 ± 0.49	2.57 ± 0.29	0.147

CON, control, basal diet; LLP, basal diet + 10^6^ CFU/kg *L. paracasei* LK01; MLP, basal diet + 10^7^ CFU/kg *L. paracasei* LK01; HLP, basal diet + 10^8^ CFU/kg *L. paracasei* LK01. Different letters in the same row indicate significant differences (*p* < 0.05). Results are expressed as mean ± standard error of the mean (SEM). *n* = 4.

## 4 Discussion

### 4.1 Effect of dietary supplementation with *L. paracasei* LK01 on performance and egg quality of laying hens

This study demonstrated that the HLP group increased average daily laying rate (ADLR) by 6.5% and reduced feed-to-egg ratio (F/E) by 8.8% compared to the CON group. Although research on *L. paracasei* in livestock and poultry production is limited, existing evidence suggests its potential to enhance production performance. Naseem et al. (29) reported that *L. paracasei*, combined with *Lactobacillus salivarius*, significantly reduced the feed-to-egg ratio in laying hens. Park et al. (30) found that 0.3% *L. paracasei* ML-7 increased feed intake and body weight in broilers, while Xu (13) showed that *L. paracasei* optimized energy allocation through enhanced intestinal fatty acid synthesis and short-chain fatty acid metabolism. As a member of the *Lactobacillaceae* family, *L. paracasei* shares mechanisms with other strains, including secretion of digestive enzymes to improve nutrient utilization and colonization of beneficial bacteria to enhance egg production and weight (31, 32). The performance improvements observed in this study align with these multifaceted effects.

Regarding egg quality, all experimental groups showed improved egg quality indices. Eggshell strength improved by 11.4% in LLP, while albumen height increased by 13.3% in HLP compared to the CON group. These findings are consistent with WeiGuo et al. (33), who reported that *Lactobacillus*-fermented feed significantly increased albumen height and Haugh units in laying hens. Morschbacher et al. (34) also observed that dietary *Lactobacillus* improved albumen quality, supporting the trends in protein-related indices observed here. For eggshell strength, Zhang et al. (35) demonstrated that *Lactobacillus plantarum* LP18 significantly enhanced eggshell strength, consistent with the significant increase (11.4%) observed in the LLP group in this study. However, the HLP group showed no significant improvement in eggshell strength but excelled in egg production rate. The differential effects between the LLP and HLP groups likely stem from dose-dependent responses. Although the underlying molecular mechanisms remain unclear and require further investigation, these results highlight the need to precisely adjust *L. paracasei* LK01 dosage based on target traits (e.g., eggshell strength or egg production) in practical applications.

### 4.2 Effect of dietary supplementation with *L. paracasei* LK01 on serum antioxidant and immune indices in laying hens

Serum biochemical indices reflect nutritional metabolism and are critical indicators of health status in laying hens. This study found no significant differences in serum AST, BUN, or Ca levels between the experimental groups (LLP, MLP, HLP) and the CON group, consistent with Liu et al. (22), suggesting that *L. paracasei* LK01 does not significantly affect core indicators of liver function, kidney function, or calcium metabolism. However, serum ALT levels were significantly elevated in the HLP group compared to the CON group, with unchanged AST levels, suggesting possible metabolic adaptation rather than liver damage, pending further investigation to confirm its mechanism.

Tissue damage triggers repair processes, with total protein (TP) supporting tissue regeneration and metabolic functions. Serum total cholesterol (TC) and triglyceride (TG) levels are key markers of lipid metabolism (36, 37). Here, TP levels increased by 26.1% in LLP, by 10.3% in MLP, and by 8.2% in HLP compared to the CON group, with LLP significantly higher than MLP and HLP. ALB levels increased by 10.0% in MLP and by 16.2% in HLP compared to the CON and LLP groups. Additionally, TC levels decreased by37.8% in MLP and by 42.7% in HLP compared to the CON group, and TG levels were reduced in all experimental groups relative to the CON group. These findings align with Yazhini et al. (38), who reported that encapsulated *L. paracasei* in broiler diets increased TP and ALB levels while reducing TC, indicating enhanc ed. protein metabolism and lipid regulation. Similarly, Xiang et al. (39) found that high-dose *Clostridium butyricum* reduced lipid levels in laying hens by upregulating fatty acid oxidation genes. The mechanism of *L. paracasei* may involve regulation of gut microbiota and bile acid metabolism (38). This modulation likely enhances calcium absorption and eggshell formation through increased short-chain fatty acid (SCFA) production and beneficial bacteria, improving egg quality. Lower serum lipid levels may also enhance egg quality, as they correlate with yolk cholesterol content (40).

Serum total antioxidant capacity (T-AOC) increased by 25.3% in LLP, by 21.7% in MLP, and by 33.3% in HLPcompared to the CON group. Malondialdehyde (MDA) levels decreased by 17.1% in MLP and by 9.4% in HLP compared to the CON group. These results are consistent with Hoffmann et al. (41), who noted the antioxidant activity of probiotics, and Gyawali et al. (42), who observed reduced serum MDA levels with *L. paracasei* in broilers. Zhao et al. (43) suggested that probiotic antioxidant effects depend on strain type, dosage, and host status. The lack of significant MDA reduction in the LLP group may reflect these factors. *Lactobacilli* enhance immune function in chickens (44–46). IL-1β, produced by macrophages, exacerbates inflammation; IL-2 promotes cytokine production and proliferation of T-helper and natural killer cells, contributing to immune responses and pathological processes (47). IL-6 induces B-cell differentiation and antibody production, serving as a marker of inflammatory damage (48). TNF-*α*, a pleiotropic cytokine, plays a key role in bacterial inhibition and inflammatory responses, with elevated levels in pathological conditions (49).

*L.paracasei LK01* supplementation did not significantly affect IL-1β levels, consistent with Mohsin et al. (50) in broilers treated with *L. plantarum*. Halder et al. (51) noted that IL-1β is typically upregulated during acute inflammation or pathogen challenge, suggesting that the absence of an immune stimulus in this trial may explain the lack of change. IL-2 levels were significantly reduced in the LLP and HLP groups. Joo et al. (23) provided a potential explanation, reporting that *L. paracasei* suppresses IL-2 secretion by increasing the proportion of regulatory T cells (Treg) in the broiler intestine, thereby reducing intestinal inflammation. Combined with the meta-analysis by Yosi et al. (52), which suggested that probiotics reduce antigen exposure by enhancing intestinal barrier integrity, it is hypothesized that the reduction in IL-2 levels may result from suppressed excessive immune activation, though the specific mechanism requires further investigation. IL-6 levels decreased by 12.0% in MLP and by 17.6% in HLP compared to the CON group, with the HLP group showing a greater reduction than the LLP group. Similarly, TNF-*α* levels decreased by 13.8% in HLP compared to the CON and LLP groups. These findings indicate selective regulation of inflammatory cytokines by *L. paracasei*, consistent with Krysiak et al. (53), who suggested that probiotics improve avian health through precise immune modulation. Xu et al. (54) and Li et al. (55) confirmed that *L. paracasei* downregulates IL-6 and TNF-α expression, improving gut health in challenged broilers, supporting the strain-specific immune effects observed here.

### 4.3 Effect of dietary supplementation with *L. paracasei* LK01 on intestinal digestive enzyme activities and cecal flora of laying hens

This study found no significant differences in digestive enzyme activities in the duodenum, jejunum, or ileum of laying hens between the experimental groups (LLP, MLP, HLP) and the CON group. This contrasts with previous reports suggesting that probiotics enhance intestinal digestive enzyme activities in poultry, promoting food catabolism and nutrient absorption (56). For instance, Wang et al. (57) reported significantly increased protease and amylase activities in the duodenum of broilers supplemented with *Bacillus coagulans*. However, many studies focus on the broader effects of probiotics on intestinal health, such as inhibiting pathogenic bacteria, enhancing intestinal barrier integrity, or modulating immunity, rather than their direct impact on digestive enzyme activities (58–60).

Although *L. paracasei* LK01 supplementation did not significantly alter intestinal digestive enzyme activities, elevated serum TP and ALB levels suggest enhanced nutrient absorption in laying hens. This indicates that *L. paracasei* may improve nutrient utilization through indirect mechanisms rather than directly affecting enzyme activities. Xu et al. (13) partially supported this, reporting that *L. paracasei* L1 indirectly enhances nutrient absorption in broilers by modulating gut microbiota metabolic pathways without altering digestive enzyme activities. This may explain the lack of significant changes in enzyme activities in this study, though further validation with metabolomics data is needed. Strain specificity and dosage are critical factors influencing probiotic effects on enzyme activity. Motlagh et al. (61) found that Bacillus. (e.g., *Bacillus subtilis*) significantly increased protease and amylase activities, whereas Lactobacillus. had a weaker effect. Yang et al. (62) demonstrated in rats that high-dose synbiotics significantly enhanced jejunal lipase and sucrase activities. The *L. paracasei* concentrations (LLP to HLP) in this study may not have reached the threshold to induce enzyme activity changes, or a longer intervention period may be required.

This experimental study showed that there was no significant difference in the indicators between the control group and each experimental group in the Alpha diversity analysis. Indicating that *L. paracasei* did not significantly alter the cecum flora, this is consistent with the findings of Liu et (22) in broiler chickens, who reported that *L. paracasei* LK01 did not significantly alter the Alpha diversity of the cecum microbiota, but significantly increased the abundance of *Bacteroides* and beneficial genera of bacteria (e.g., *Ruminococcaceae*, *Lachnospiraceae* and *Faecalibacterium*) abundance. In contrast, this study observed a decrease in the abundance of specific pathogenic bacteria rather than an increase in beneficial bacteria in laying hens, possibly reflecting differences in microflora response between laying hens and broilers.

At the phylum level, *Spirochaetota* abundance was significantly reduced in the experimental groups. Pathogenic *Brachyspira* within *Spirochaetota* causes avian intestinal spirochaetosis (AIS), leading to diarrhea and reduced egg production. *Brachyspira intermedia*, a primary AIS pathogen in laying hens, impairs intestinal mucosa and nutrient absorption (63, 64). Thus, the significant reduction in *Spirochaetota* abundance may lower the risk of intestinal inflammation. In addition, *Bacteroides*, a key degrader of polysaccharides and dietary fiber, produces short-chain fatty acids (SCFAs) that serve as energy sources for intestinal epithelial cells and support barrier function and immune modulation (65).

At the genus level, *Bacteroides* and *Rikenellaceae RC9 gut group* were the dominant cecal bacteria in laying hens, consistent with Joo et al. (23), who reported increased Bacteroides abundance in broilers supplemented with *L. paracasei* NSMJ56. This increase may result from SCFA production (e.g., propionic acid) by *L. paracasei*, promoting beneficial bacteria. Notably, *Fusobacterium* abundance was significantly reduced in the MLP and HLP groups, and *Treponema* abundance was significantly lower in all experimental groups. While some *Fusobacterium* strains (e.g., *F. mortiferum*) produce bacteriostatic substances (66), their overproliferation is linked to intestinal barrier damage (67). *Treponema hyodysenteriae* has been shown to induce cecal mucosal inflammation and diarrhea in chickens (68), so its reduced abundance may be a key mechanism by which *L. paracasei* enhances gut health. *In vitro* studies showed that *L. paracasei* significantly reduces *Salmonella* adhesion and invasion while downregulating its virulence gene expression (69), consistent with the reduced abundance of pathogenic genera observed here. The current conclusion relies primarily on alpha diversity and specific taxonomic shifts; future studies should incorporate beta-diversity analyses (e.g., PCoA) to comprehensively evaluate overall community structure and employ PICRUSt or shotgun metagenomics to elucidate functional mechanisms underlying taxonomic changes, such as virulence pathway regulation.

## 5 Conclusion

Dietary supplementation with *L. paracasei* LK01 enhances laying hen performance and egg quality, improves serum biochemical indices, increases serum antioxidant capacity, and suppresses harmful cecal microbiota. The results of this study suggest that the optimal dietary concentration of *L. paracasei* LK01 for laying hens is 10^8^ CFU/kg. However, as 10^8^ CFU/kg was the highest dose tested, further studies are needed to confirm the optimal concentration. While the sampling strategy was sufficient for detecting significant effects, future studies could increase biological replicates for intestinal analyses to further validate findings.

## Data Availability

The 16S rRNA sequencing datasets for this study can be found in the PRJNA1313457 of NCBI Sequence Read Archive (SRA) [http://www.ncbi.nlm.nih.gov/bioproject/1313457].

## Appendix

**Table A1:** Information on all the kits used to measure serum biochemicals and intestinal digestive enzymes in laying hens.

IgA	IgA ELISA kit	H108-1-2
IgM	IgM ELISA kit	H109-1-1
IgG	IgG ELISA kit	H106-1-1
T-SOD	Total superoxide dismutase (SOD) assay kit	A001-1-1
GSH/Px	Glutathione peroxidase (GSH-Px) kit	A005-1-1
T-AOC	Total antioxidant capacity (T-AOC) kit	A015-2-1
MDA	Malondialdehyde (MDA) kit	A003-1-2
IL-1β	Interleukin-1β assay kit	H002-1-2
IL-2	Interleukin-2 assay kit	H003-1-1
IL-6	Interleukin-6 assay kit	H007-1-1
TNF-α	TNF-α	H009-1-1
Protease (IU/mL)	Protease assay kit	A080-2
Lipase (U/mL)	Lipase (LPS) assay kit	A054-1
Amylase (U/L)	Total amylase (T-AMY) assay kit	A054-2
DNA extraction	E.Z.N.A.® Soil DNA Kit	D5625-01
PCR amplification	TransStart FastPfu DNA polymerase kit	AP221-02
Library preparation	Nextera XT DNA library preparation kit	FC-131-1096
